# Experiences of healthcare practitioners providing nutrition care to people with cancer in New Zealand: A qualitative study

**DOI:** 10.1177/02601060231207439

**Published:** 2024-01-27

**Authors:** Rana Peniamina, Rachael Mira McLean

**Affiliations:** Department of Preventive and Social Medicine, Dunedin School of Medicine, University of Otago, Dunedin, New Zealand

**Keywords:** Nutrition, cancer, qualitative, New Zealand, healthcare

## Abstract

**Aim:** To explore the perspectives and experiences of healthcare practitioners in providing nutrition care to people with cancer in New Zealand. **Methods:** Semi-structured interviews were conducted with 16 New Zealand healthcare professionals (HCPs) practicing in specialist secondary and tertiary cancer care (both public and private) who had previously completed an online survey about nutrition in cancer care. Interviews were recorded and transcribed verbatim, and thematic analysis was undertaken. **Results:** Participants identified nutrition as important in cancer care, from diagnosis to survivorship, in order to maximise recovery and ongoing health. While participants reported that the best person to provide high-quality individualised nutrition care is a dietitian, other HCPs also have a role in nutrition care. Limited nutrition care is currently available, especially for those in rural areas, which impacts health and equity in cancer care. Participants identified a need for increased dietitian capacity in the workforce as well as a need for nutrition resources that were accessible and appropriate. **Conclusion:** The perspective of participants was that more must be done to provide nutrition care as part of cancer care in New Zealand to improve health and wellbeing among people with cancer.

## Introduction

Cancer is a leading cause of death and disability in New Zealand and people with cancer are increasingly living longer as treatments improve (Te Aho o Te Kahu, 2021). International guidelines recommend that nutrition care is an essential component of cancer care from diagnosis, through treatment and through survivorship to optimise health and quality of life (Arends et al., 2017; Caccialanza et al., 2022; World Cancer Research Fund/American Institute for Cancer Research 2018). For the purpose of this study, we have defined nutrition care as all diet and nutrition-related advice, information, assessment, diagnosis, support, interventions, monitoring and evaluation that might be provided to aid patients’ health and wellbeing. Healthcare professionals (HCPs) should provide advice on food and nutrition and support patients to meet their nutritional needs, to prevent malnutrition and cachexia during cancer treatment, to help manage symptoms such as nausea and vomiting, difficulty swallowing, constipation or diarrhoea and to manage a range of other complex nutrition needs that are specific to cancer type, stage or individual patient needs (Arends et al., 2017; Caccialanza et al., 2022). Optimal nutritional status improves treatment tolerance, outcomes and quality of life among those undergoing treatment for cancer (Arends et al., 2017; Caccialanza et al., 2022; Schwedhelm et al., 2016; World Cancer Research Fund/American Institute for Cancer Research 2018). Long-term, optimal nutrition reduces subsequent risk of other non-communicable diseases (NCDs) such as diabetes and cardiovascular disease (Mozaffarian, 2016), as well as reducing the risk of cancer recurrence (Blackwood et al., 2020; Schwedhelm et al., 2016).

In New Zealand, at the time of data collection, the publicly funded healthcare system was divided into 20 regional District Health Boards (DHBs) where the majority of specialist multidisciplinary cancer treatment (inpatient and outpatient) occurred. All DHBs provided some surgical treatment for cancer, but some treatments were only available at one of the six specialist centres in the larger cities. There were also four private cancer treatment centres that provide treatment to a smaller proportion of the population: those with private health insurance, and/or able to privately fund treatment (Te Aho o Te Kahu, 2021).

Previous research in New Zealand and internationally shows that people with cancer would like advice from HCPs on diet and nutrition that is evidence-based and appropriate to their cultural and sociodemographic context (Keaver et al., 2022b; Koutoukidis et al., 2018; Peniamina et al., 2021). Those who do not get adequate information from HCPs may turn to advice from other sources (such as online), which may not be evidence-based (Antheunis et al., 2013; Peniamina et al., 2021). Results from a recent survey of New Zealand HCPs (including medical oncologists, radiation oncologists, nurses, surgeons and dietitians) showed that nutrition was considered important for both cancer recovery and overall patient wellbeing and indicated strong support for including nutrition care in cancer care (Peniamina and McLean, 2022). However, the survey also showed that many HCPs feel they are not adequately trained to provide this advice (Peniamina and McLean, 2022). In addition, access to care from a dietitian was mostly limited to patients who were malnourished or at high risk of malnutrition during treatment (Peniamina and McLean, 2022). The current study aimed to further explore the perspectives and experiences of healthcare practitioners in providing nutrition care to people with cancer in New Zealand, with a particular focus on identifying and understanding gaps in the current nutrition care and what resources would be helpful to better meet the nutrition care needs of people with cancer. This served to support the overall goal of identifying ways to improve access to cancer-specific nutrition information and care for cancer patients and cancer survivors.

## Methods

This study was approved by the University of Otago Human Ethics Committee (Health), approval number H20/036, and has been performed in accordance with the ethical standards laid down in the Declaration of Helsinki. All participants gave their written informed consent prior to their inclusion in the study. Consolidated criteria for reporting qualitative research (COREQ) guidelines (https://www.equator-network.org/reporting-guidelines/coreq/) for reporting were used in this study.

Participants were a subgroup selected from a group of New Zealand HCPs practicing in specialist secondary and tertiary cancer care (both public and private) who had previously completed an online survey about nutrition in cancer care (Peniamina and McLean, 2022) and had indicated their availability for a follow-up interview. Forty-six (41%) of the survey participants had indicated possible availability for an interview. A purposeful sample was selected from that group, to include both male and female participants and to ensure representation from different professions, regions (including urban and rural settings) and both public healthcare (where most cancer care is provided) and private cancer care settings. This allowed us to access and understand a wider range of perspectives and experiences among different HCPs in different contexts of cancer care. Potential participants identified via this process were contacted by an email inviting them to participate in an interview. Of those, four did not respond and two indicated they were not available due to time constraints. The process of emailing potential participants and completing interviews continued until data saturation was reached. We defined data saturation as the point where “the researcher can be reasonably assured that further data collection would yield similar results” (Faulkner and Trotter, 2017).

All participants (n = 16) took part in semi-structured videoconference (using Zoom platform) interviews with the same experienced qualitative interviewer/researcher (RP). Videoconferences allowed the interviews to proceed during COVID-related restrictions on travel and in-person contact without losing access to body language and facial expressions that are part of a face-to-face interview interaction and add to the interviewer's understanding of what the participants are saying (Saarijärvi and Bratt, 2021). Data collection were completed between November 2020 and February 2021.

Interviews started with an explanation of the aims of the study. Participants were asked about their perspectives and experiences of providing nutrition information and care for cancer patients in their care (see Table 1 for the interview guide). The questions were developed based on the findings from the survey about nutrition in cancer care (Peniamina and McLean, 2022) and a prior qualitative study with cancer survivors (Peniamina et al., 2021). While all the main questions were asked during each interview, the semi-structured format for the interviews allowed for sub-questions and prompts to be used as needed and for question order to vary to follow the natural flow of conversation within the interview. Interviews lasted between 21 and 52 min (average 32 min). The interviews were audio-recorded (with consent), transcribed verbatim using a transcription service, and checked for accuracy by the researchers. To improve the flow/readability of the quotes used in this publication, the authors have removed filler words (e.g., um, ah, you know) and repeated words.

**Table 1.** Interview guide

Main questions	Sub-questions and prompts
What do you consider the important aspects of diet and nutrition for cancer patients?	- Why/how? - How does nutrition fit into cancer prevention/recovery? - How important is it for cancer patients? Why? - Any specific needs related to type of cancer?
In an ideal world, what diet/nutrition-related information and support do you think cancer patients should have access to?	
How do you feel about providing dietary advice/support to cancer patients as part of your role?	- Why/explain - What makes it harder/easier?
What is the standard process (or guideline), if any, regarding provision of diet/nutrition advice and support to cancer patients?	- Is that the standard for this District Health Board/region? - What about access to recommended foods in hospital or at home?
Can you tell me about the diet/nutrition resources you have access to that you can share with patients?	- What sources do you use for information about diet, nutrition and cancer? Can you explain your reasons for using these sources? - Are these specific to cancer? What is their focus? (e.g., all cancers/specific cancers, symptom/side-effect management, cancer recovery, diet/nutrition recommendations for cancer recovery/prevention) - What other diet or nutrition information and support is available to your patients? (pamphlets, websites, support groups, ongoing dietitian support, …) - What about other practitioners within your clinic (do they offer diet-related support)? - What about referrals for dietary advice/support outside this clinic? - How do patients access these services? - What about cultural differences? What support/resources are available to meet the needs of different cultural groups? e.g., in native language, or recommendations that fit with cultural food norms - Is there any financial support available for cancer patients in this region (e.g., for access to recommended food and/or diet/nutrition support)?
If more resources about diet/nutrition and cancer were made available in New Zealand, how comfortable would you feel about directing patients to those resources?	- Explain reasoning

Thematic analysis (Braun and Clarke, 2006) of the interview transcripts was completed by both authors. RP is a qualitative researcher with expertise in food science and nutrition. RM is a medical practitioner with expertise in mixed methods research in public health and nutrition. The thematic analysis process followed a series of steps:

1. Familiarisation with the data (RP interviewed the participants and checked the accuracy of the transcripts, followed by reading and re-reading the transcripts while looking for patterns and meaning in the text. RM read the transcripts once they had been checked).
2. Assigning initial codes (RP and RM assigned descriptive code names for identified patterns in the data and selected and saved relevant sections of text under each code).
3. Searching for themes (RP sorted the initial codes into potential themes and subthemes, RM reviewed codes from RP and themes were discussed by both authors).
4. Reviewing themes (RP and RM checked themes for coherence and clarity, and recoded text that did not fit the theme).
5. Naming themes (RP and RM identified names that describe the essence of each theme).

A pragmatic approach was taken in the thematic analysis to specifically build our understanding of the nutrition care available to people with cancer, barriers and enablers, and what resources (e.g., information and types of care) are likely to be useful. This involved identifying common themes from the data (as relevant to the research aims), grouping passages within those themes and paying attention to both similar and contrasting perspectives within the themes. Manual coding was completed with the aid of the NVivo 12 software package (QSR International 2018). Representative quotes were identified and annotated with a participant number and profession category (Medical Specialist: MS, Nurse/Support worker: N/SW, Dietitian: D).

## Results

The 16 interview participants (75% female) included 7 medical specialists representing different areas of cancer care (medical oncologist, radiation oncologist, surgeon, haematologist), 5 dietitians (including two specialist oncology dietitians), 3 cancer care nurses and 1 cancer support worker. The participants represented 13 of the 20 DHB areas in New Zealand, including both urban and rural/remote areas, public and private practices, and all the main oncology centres.

Four main themes were identified from the thematic analysis:

1. Nutrition care is important in cancer care from diagnosis to survivorship.
2. Dietitian is the expert, but others have a role.
3. Limited nutrition care available, with likely impacts on health and equity.
4. A way forward-funding, workforce development and resources.

### Nutrition care is important in cancer care from diagnosis to survivorship

Nutrition was described as “comprehensively important” (MS1) for people with cancer and as “integral” to their care and for survivorship (MS4). Participants reported that nutrition care should be offered throughout the cancer journey, from pre-treatment (to ensure that people with cancer are well enough for treatment and to maximise the success of cancer treatment) through to the end of cancer care to prevent recurrence and minimise the risk of morbidity of other NCDs such as cardiovascular disease.

Participants identified a range of specific areas where nutrition care was important, depending on patients’ individual circumstances, including maintaining weight (particularly muscle mass) for patients with malnutrition and cachexia, management of gastrointestinal symptoms, reducing fat mass to limit inflammation (for prevention), ensuring food safety (for immunocompromised patients), maintaining dietary sufficiency while experiencing side effects (such as difficulty swallowing, nausea, diarrhoea), help with optimising nutrition while under financial pressure and advice with nutritional supplementation where necessary. However, it is important to note that “what's important depends on the context of the patients” (MS7).

In terms of reducing risk … reducing fat mass because there's where a lot of the inflammation…. (D2)

On a regular basis, we have people who struggle financially to put just food, be it healthy or not healthy on the table. (N/SW2)

The patients we treat, some of them become profoundly neutropenic. So their white cells get wiped out by the chemotherapy. That is quite common in hematology protocols, including stem cell transplant. And so, for that group, there is an issue around safety of food. (MS6)

Participants emphasised that good nutrition was important for minimising treatment toxicity and side effects and was likely to result in better treatment outcomes.

I think it's fundamental to treatment. It can enhance treatment and, without good nutrition, treatments will fail to be as effective. (D1)

They need adequate nutrition to reduce the side effects, the toxicity from the treatment, and also to moderate effects of the cancer. (MS3)

Participants also talked about the importance of healthy eating for ongoing health after treatment in terms of preventing cancer recurrence and/or other NCDs, as well as for prevention of primary cancer and other NCDs in the general population.

It's a huge part of cancer prevention. The research is very clear on that. (D5)

Good knowledge about what is a healthy diet in the context of cancer, which would fortunately also protect for cardiovascular [disease], I think is important for the general population. … It certainly helps their recovery from the treatment and then sort of that broader thing of prevention and long term prevention of recurrence (MS1)

I do see nutrition as an important part of prevention in general for people that haven’t had cancer before, but also for people that have it. (D4)

### Dietitian is the expert, but others have a role

Participants (including non-dietitian HCPs) identified dietitians as the experts in provision of nutrition care and many supported the view that every person with cancer should get “individualized care from a skilled dietitian” (D1).

I would like to see every cancer survivor in New Zealand have an opportunity to speak with a dietitian. (MS5)

Participants reported that other health professional groups also had a role in provision of nutrition care (e.g., introducing the importance of nutrition and providing some introductory information). However, there was some concern that non-dietitian HCPs may not be adequately qualified to provide the best nutrition care. Participants who were not dietitians reported that they lacked the expertise or capacity to provide adequate nutrition care for their patients.

I see my role as introducing the importance of it, explaining aspects of it, but I do believe it should be supported by somebody who’s more expert than I am. (MS1)

I’ll tell people the basics about nutrition, but I certainly am no expert. (N/SW3)

### Limited nutrition care available, with impacts on health and equity

Access to care from a dietitian was only available to a limited number of people undergoing cancer treatment. These included people identified as high risk of malnutrition due to cancer type or treatment (such as those with head and neck cancers) and people with identified malnutrition according to specific screening criteria (e.g., using the Malnutrition Screening Tool; Ferguson et al., 1999). Nutrition care provided therefore tended to focus on ensuring adequate calorie intake to maintain weight. However, even high-risk patients were often not seen by dietitians until weight loss and malnutrition were already clearly evident.

I just deal with a tiny fraction of the people who could be helped. Every week I get referrals and I say, “No, I can’t see them.” I just see the top priority ones. … I’m just seeing the people who really, who need to be artificially fed in some way. Almost always. Because anyone else, they’re not prioritized according to the Ministry of Health triage system. (D1)

We do have access to the hospital dietitians, but they’re mostly used for the inpatients who are in trouble [malnourished], if that makes sense. (MS6)

With access to dietetic care being mostly limited to patients with malnutrition or at high risk of malnutrition, other patient groups who could benefit from nutrition care (not related to malnutrition) were missing out.

Unless they’re head and neck or colorectal, they don’t tend to get that support [access to dietitian] as much. (N/SW4)

If you’re just an outpatient, and you want some dietitian support, or in the community [outside of inpatient hospital-based cancer care] … You just have to wait. The waiting list is quite a long time, and the priorities … they may not even see your patient. (MS4)

Participants explained that some cancers/treatments result in ongoing unpleasant symptoms, weight gain or increase risk for later health problems (e.g., metabolic disorder) and affected patients would benefit from dietetic input.

And all the while they’re having problems with discomfort in their abdomen at night because of foods that they don’t tolerate that they use- and “what should I avoid?” And just a half an hour discussion about that can affect their lives hugely. And they can live comfortably for the rest of their life. (D2)

Then there's another group of patients who are actually on hormone treatment, long-term hormone treatment that changes their metabolism and they tend to actually put on weight, they become obese. … I think there's a kind of big unmet need, is these, big population of [patients] who are having, because of the treatment, they tend to put on weight and then find it hard to lose weight. (MS3)

Several participants discussed the need for continued nutrition care for cancer survivors post-treatment.

You know, “what do I do once I’ve got through my treatment?”, “I’ve been cured, and I’ve come out the other side. What should I eat, what should my lifestyle look like?” You know, “what things can I do to help myself stay free of cancer?” I get asked that question day in and day out and I think that is a massive need for people who are cancer survivors. You know, they need information on what to do now. (MS5)

Participants identified some important inequities that resulted in limited access to specialist nutrition care. For example, rural services had limited resources to be able to reach patients in more remote settings.

Because of my limitations in FTE [Full Time Equivalent working hours] in our big region for being a small DHB [District Health Board], I don’t get to see every person … the rural patient gets quite often missed in that. So they would only be referred to a dietitian by the time they have a problem [are already very malnourished]. (N/SW2)

A lack of culturally appropriate printed resources was identified as a barrier to providing adequate nutrition care to a culturally diverse population.

Some places in Australia that have got good [resources]- and we kind of score out the things that- of the foods or the words that are different for here. They’re very kind of Pākehā [European]. (D1)

Although some clinics were able to provide written resources (e.g., pamphlets) to patients who were unable to access specialist dietetic care, these were not considered adequate for those with limited financial resources, or those with low literacy. Participants emphasised the importance of individualised dietetic care for equitable care.

A lot of people aren’t very good at reading. But I find that if I sit down with them and go through things … It's good if it's something you can kind of go through together, not just give people a piece of paper. That's actually in a sense, quite a fob off [attempt to appease someone with something of lower quality or different to what they need] to do that. (D1)

### A way forward: funding, workforce development and resources

Participants wanted increased access to individualised care from a dietitian for people with cancer. Post-treatment access to dietitians in the community was also seen as important in order to improve survival and lessen the risk of other NCDs.

I think they should have access to reliable information that is individualized to them, because unfortunately the internet is filled with all sorts of cancer prevention or cancer healing information to do with nutrition and it can be really confusing for patients. (D4)

In the ideal world, is if there was an oncology dietitian who could actually, the same as an oncology social worker or nurse, could actually meet [cancer patients] and do an individualised plan with every patient. (N/SW4)

I would like to see every cancer survivor in New Zealand have an opportunity to speak with a dietitian who has experience in the sort of diet that might be able to be followed that may mean that that patient has a better chance of living a longer, healthier life going forward. (MS5)

Participants also identified a need for consistent New Zealand-specific culturally appropriate cancer nutrition resources for both HCPs and patients to use. These could be in multiple formats, including online and paper-based, written and audio/video format, instructive and interactive. Ideally, these would be updated regularly according to new evidence and include training modules for HCPs.

It’ll be really cool to have something. Like I am scrambling on the Internet and different places finding information that I share with my patients, but it would be really nice to have something New Zealand based that we’re all giving the same advice across the country. (N/SW3)

Having a national resource where we all just… we’re getting the same advice, would be nice. I would certainly find that, for me as a professional, to have something that is directed to professionals, as a way of making it easier to keep your knowledge at that mid-level, but also then resources for patients. Yeah, that would be very, very helpful. (MS1)

## Discussion

This qualitative study shows that HCPs involved in cancer care in New Zealand view the current availability of dietetic and nutritional care for people with cancer as limited and think an increase in the availability of nutrition care would benefit many people with cancer. Dietitians were identified as the best person to provide this care, although participants saw other HCPs as also having a role. Participants identified the need for nutritional advice and care throughout the cancer care journey, from diagnosis to survivorship, in order to maximise health. However, currently, dietetic care is only available to people who are identified as malnourished, those with specific cancers such as head and neck cancer, or those who can afford private care. This results in sub-optimal care and will contribute to inequities in cancer care and outcomes. Barriers to provision of adequate nutritional care include inadequate numbers of dietitians in tertiary cancer care settings, restrictive referral policies, and lack of time and expertise for other HCPs (doctors, nurses, and other cancer support workers). Although individualised face-to-face contact with a dietitian was seen as the best option, participants also identified the need to address the current lack of useful New Zealand-specific and culturally appropriate resources. This lack of appropriate resources is likely to contribute to inequities in nutritional care of people with cancer.

These results support the findings from our survey of New Zealand HCPs (Peniamina and McLean, 2022) and our interviews with cancer survivors (Peniamina et al., 2021), and add more detailed information on what additional care and/or resources are needed and why. In addition, similar findings have been reported in other countries. For example, time and lack of in-depth nutrition expertise were discussed as barriers to dietary discussions by HCPs in the USA (Smith et al., 2016) and the UK (Koutoukidis et al., 2018)

There is clear evidence of health benefits of good nutritional status throughout cancer treatment and beyond, and that accessible evidence-based nutritional care for people with cancer is essential to ensure health equity in cancer care and outcomes (Blackwood et al., 2020; Caccialanza et al., 2022; Schwedhelm et al., 2016; World Cancer Research Fund/American Institute for Cancer Research 2018). Current guidelines and evidence reviews recommend nutritional care from appropriately qualified healthcare practitioners during cancer treatment (Arends et al., 2017; Caccialanza et al., 2022; World Cancer Research Fund/American Institute for Cancer Research 2018). Consistent with the findings of our earlier research with cancer survivors (Peniamina et al., 2021), international research shows that people with cancer want advice and care regarding food and nutrition in multiple formats, for example, face-to-face, written, and/or interactive online formats (Keaver et al., 2022a; Pugh et al., 2018; Virdun et al., 2020). Information must be appropriate to the age and cultural background of the person with cancer, as well as to their symptoms and nutritional needs. Further, some studies suggest that a diagnosis of cancer can be a trigger for healthy behaviour change in individuals and families, so provision of evidence-based nutrition care can have broad benefits, beyond those relating to cancer treatment and recovery (Pugh et al., 2018; Williams et al., 2013).

Other New Zealand-based research has also highlighted the need for written resources that are culturally appropriate in various settings, including for dialysis patients (McLean et al., 2021; Xie et al., 2022) and in food purchasing settings (Eyles et al., 2009). Written resources should be relevant to patients’ cultural background, including pictures of cultural foods, and availability in relevant languages (Eyles et al., 2009).

This research has several strengths. Interviews were conducted with HCPs in a range of different roles in cancer care, who worked in urban and rural as well as public and private settings. These HCPs were selected from a larger survey sample (n = 112) and the views expressed in this qualitative study were consistent with the survey findings (Peniamina and McLean, 2022), although this was not formally triangulated. This allowed us to gain understanding of a range of experiences and perspectives, as influenced by cancer care specialty and different contexts. Another key strength was the semi-structured approach in the interviews which allowed participants room to discuss what they believed was important rather than be limited by the nature of the questions asked. This research also has several limitations. We identified participants from a range of professional groups, however, we did not aim to have a representative sample. The views of the participants of this study are not necessarily representative of HCPs as a whole. In fact, given the nature of the research, it is likely that HCPs with a particular interest in nutrition were more likely to participate so the views expressed may overstate the degree to which HCPs value nutrition care as part of involvement in cancer treatment in New Zealand.

In conclusion, our research shows that HCPs believe that more must be done to provide nutrition care as part of cancer care in New Zealand. This should include expanding the capacity and availability of oncology-specialised dietitians in cancer care, education of other HCPs in nutrition specific to cancer care, and development of culturally appropriate New Zealand-specific resources. Many participants suggested that an online nutrition resource for HCPs and patients would be useful. Expanding access to nutrition care has the potential to enhance health and equity in cancer care in New Zealand.
